# Reproducibility of ^19^F‐MR ventilation imaging in healthy volunteers

**DOI:** 10.1002/mrm.28660

**Published:** 2021-01-28

**Authors:** Benjamin J. Pippard, Mary A. Neal, Adam M. Maunder, Kieren G. Hollingsworth, Alberto Biancardi, Rod A. Lawson, Holly Fisher, John N. S. Matthews, A. John Simpson, Jim M. Wild, Peter E. Thelwall

**Affiliations:** ^1^ Newcastle Magnetic Resonance Centre Newcastle University Newcastle upon Tyne United Kingdom; ^2^ Translational and Clinical Research Institute Newcastle University Newcastle upon Tyne United Kingdom; ^3^ POLARIS, Department of IICD University of Sheffield Royal Hallamshire Hospital Sheffield United Kingdom; ^4^ Respiratory Medicine Sheffield Teaching Hospitals National Health Service Foundation Trust Sheffield United Kingdom; ^5^ Population Health Sciences Institute Newcastle University Newcastle upon Tyne United Kingdom; ^6^ School of Mathematics, Statistics and Physics Newcastle University Newcastle upon Tyne United Kingdom; ^7^ Respiratory Medicine Newcastle upon Tyne Hospitals National Health Service Foundation Trust Newcastle upon Tyne United Kingdom

**Keywords:** ^19^F‐MRI, lung, perfluoropropane, reproducibility, ventilation

## Abstract

**Purpose:**

To assess the reproducibility of percentage ventilated lung volume (%VV) measurements in healthy volunteers acquired by fluorine (^19^F)‐MRI of inhaled perfluoropropane, implemented at two research sites.

**Methods:**

In this prospective, ethically approved study, 40 healthy participants were recruited (May 2018‐June 2019) to one of two research sites. Participants underwent a single MRI scan session on a 3T scanner, involving periodic inhalation of a 79% perfluoropropane/21% oxygen gas mixture. Each gas inhalation session lasted about 30 seconds, consisting of three deep breaths of gas followed by a breath‐hold. Four ^19^F‐MR ventilation images were acquired per participant, each separated by approximately 6 minutes. The value of %VV was determined by registering separately acquired ^1^H images to ventilation images before semi‐automated image segmentation, performed independently by two observers. Reproducibility of %VV measurements was assessed by components of variance, intraclass correlation coefficients, coefficients of variation (CoV), and the Dice similarity coefficient.

**Results:**

The MRI scans were well tolerated throughout, with no adverse events. There was a high degree of consistency in %VV measurements for each participant (CoV_observer1_ = 0.43%; CoV_observer2_ = 0.63%), with overall precision of %VV measurements determined to be within ± 1.7% (95% confidence interval). Interobserver agreement in %VV measurements revealed a high mean Dice similarity coefficient (SD) of 0.97 (0.02), with only minor discrepancies between observers.

**Conclusion:**

We demonstrate good reproducibility of %VV measurements in a group of healthy participants using ^19^F‐MRI of inhaled perfluoropropane. Our methods have been successfully implemented across two different study sites, supporting the feasibility of performing larger multicenter clinical studies.

## 1 INTRODUCTION

Magnetic resonance imaging is an attractive approach to the investigation of respiratory disease, given its noninvasive nature and lack of exposure to ionizing radiation. Conventional MRI of the lungs remains challenging, however, due primarily to the low proton density of lung tissue and magnetic field inhomogeneities that exist at ubiquitous air–tissue interfaces.^1^ The use of exogenous gas agents can overcome these challenges by direct visualization of inhaled gas within the lungs, enabling assessment of regional ventilation properties. Specifically, hyperpolarized‐gas MRI (HP‐MRI) is well established in research settings^2^, ^3^ and has led to the development of novel imaging biomarkers relating to lung function.^4^, ^5^, ^6^ Of these, the percentage ventilated lung volume (%VV) and related ventilation defect percentage have been reported widely,^7^, ^8^ providing clinically useful metrics of ventilatory function that correlate strongly with conventional spirometric indices.^9^, ^10^ Previous studies have established the reproducibility of these HP‐MRI measurements in healthy volunteers^7^, ^9^, ^11^ and patients with respiratory disease,^11^, ^12^, ^13^, ^14^ showing improved sensitivity to early smoking‐related disease^15^ and asthma control^16^ compared with spirometry. However, the requirement for specialized polarizing equipment and expertise remains a potential barrier to widespread clinical adoption of this technique.

The ^19^F‐MRI of inhaled perfluoropropane (PFP) offers an alternative approach to human lung imaging, with potential to provide similar functional information regarding pulmonary ventilation to HP‐MRI.^17^ Crucially, this technique uses an inert gas that can be mixed with oxygen and imaged at thermal polarization, thus avoiding the need for hyperpolarization. A small number of studies have demonstrated feasibility of this method to assess regional gas distribution in healthy volunteers^18^, ^19^, ^20^, ^21^ and patients with respiratory disease,^19^, ^22^, ^23^, ^24^ building on an extensive body of preclinical work.^25^, ^26^, ^27^, ^28^, ^29^, ^30^, ^31^, ^32^ The ability to breathe PFP over the course of several respiratory cycles offers further scope to acquire dynamic measurements of gas distribution, which has recently shown promise for evaluating airflow limitation in chronic obstructive pulmonary disease.^22^, ^33^ Nonetheless, pulmonary ^19^F‐MRI remains in relative infancy, and, to date, no studies have evaluated the utility of %VV measurements using this approach. Determining the capability of static ^19^F‐MR ventilation imaging to accurately report on such lung biomarkers in healthy volunteers is a necessary precursor to performing downstream patient studies. Moreover, the implementation of ^19^F‐MRI scan procedures at more than one study site serves to define a baseline standard for robust application of future dynamic ^19^F‐MRI protocols.

The purpose of this study was to assess the utility of static ^19^F‐MR ventilation imaging by determining the interscan and interobserver reproducibility of %VV measurements in healthy participants across two research sites.

## 2 METHODS

This prospective, dual‐center study was approved by the local research ethics committee (Ref 16/NE/0282) and the National Health Service Health Research Authority.

### 2.1 Study population

Forty healthy participants (21 males, 19 females; ages 23‐67 years, mean = 41) provided written informed consent and were screened for eligibility at one of two UK study sites (20 at Newcastle [site A]; 20 at Sheffield [site B]) between May 2018 and June 2019. Participants were recruited from local university and health care institutions at respective study sites through poster and/or email advertisement. All participants were nonsmokers in good health, with normal lung function as assessed by spirometry.^34^ Study inclusion and exclusion criteria are summarized in Table 1.

**TABLE 1:** Summary of study participant eligibility criteria

Inclusion criteria	Exclusion criteria
Age 18 years or older Normal spirometric function^a^ Body weight 50‐100 kg	History of respiratory disease and/or current evidence of respiratory tract infection. Cardiac or cerebrovascular disease, anemia, or other serious medical condition. Current prescribed medication (excluding oral contraceptive). History of smoking in past 2 years: ex‐smoker with greater than 2‐pack year history^b^ and/or ex‐smoker who has smoked for more than 2 years in total. Contraindications to MRI (including incompatible body habitus/BMI greater than 35). Pregnant or breastfeeding.

Abbreviations: BMI, body mass index; FEV_1_, forced expiratory volume in 1 second; FVC, forced vital capacity.

^a^ FEV_1_ greater than or equal to 80% predicted; FEV_1_/FVC greater than or equal to 0.7.

^b^ 2‐pack year refers to smoking 40 cigarettes per day for 1 year (1 pack = 20 cigarettes).

### 2.2 Magnetic resonance imaging

Participants underwent a single MRI scan session at one of the two study sites. All scans were performed supine using a Philips Achieva (site A) or Philips Ingenia (site B) 3T scanner (Philips Healthcare, Guildford, United Kingdom) interfaced to a ^19^F/^1^H chest birdcage coil (Rapid Biomedical, Rimpar, Germany). Anatomical ^1^H scans were acquired after instructing participants to perform a breath‐hold at maximal inspiration, using a 3D spoiled gradient‐echo sequence (Table 2).

**TABLE 2:** Summary of scan parameters for ^1^H and ^19^F MR acquisitions

Parameter	Scan		
^1^H anatomical	^19^F FID	^19^F ventilation
TE (ms)	0.49	—	1.7
TR (ms)	4.0	200	7.5
Flip angle (^o^)	6	90	45
FOV (mm^3^)	440 × 440 × 247.5	—	400 × (310‐360) × 250
Resolution (mm^3^)	3 × 3 × 7.5	—	10 × 10 × 10
Matrix size (voxels)	147 × 147 × 33	—	40 × (31‐36) × 25
Bandwidth (Hz/pixel)	3400	—	500
Number of averages	1	50	3
Acquisition time (s)	14.6	10	13.4
Number of samples	—	256	—
Sampling frequency (Hz)	—	8000	—

**FIGURE 1:**
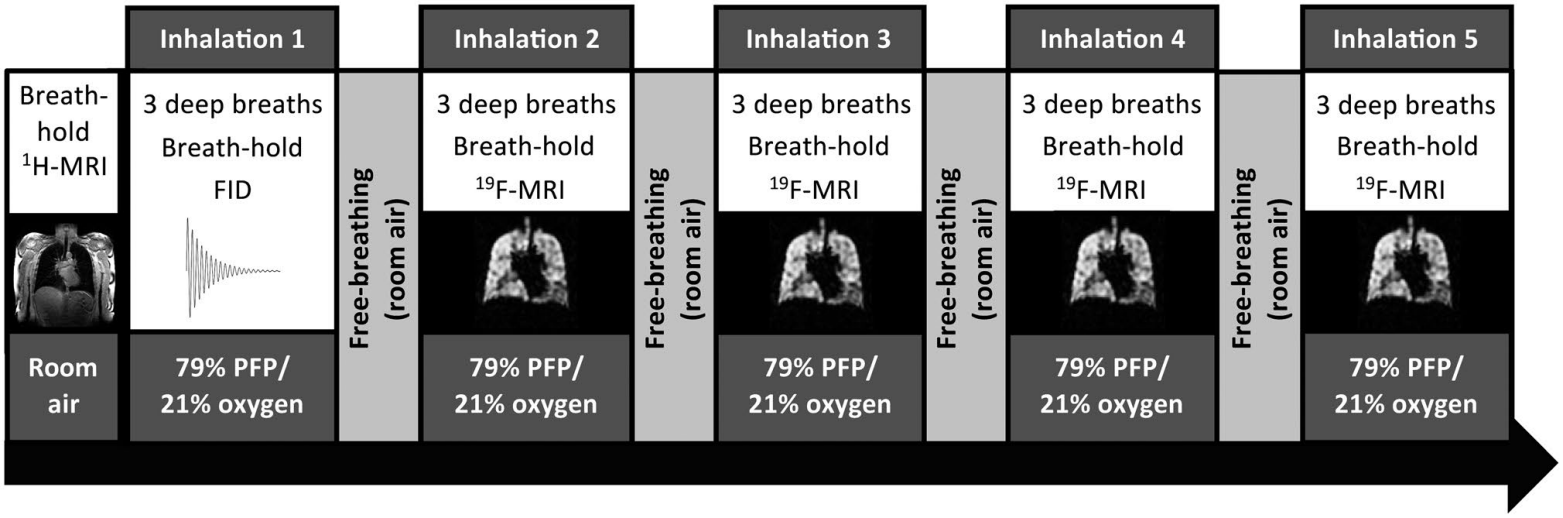
Magnetic resonance imaging protocol, consisting of an initial anatomical ^1^H‐MRI breath‐hold acquisition followed by five fluorine (^19^F)‐MRI breath‐hold acquisitions. The first perfluoropropane (PFP) gas inhalation session was used for a whole‐lung spectroscopy scan; the remaining four inhalation sessions were used to acquire ^19^F‐MR ventilation images, each separated by an interval of approximately 6 minutes

Participants were subsequently instructed to inhale a 79% PFP/21% oxygen gas mixture (BOC Special Products, Guildford, United Kingdom) on five occasions during the MRI scan session (Figure 1). Each gas inhalation session lasted approximately 30 seconds, consisting of three deep breaths of gas from a starting point of relaxed end‐expiration, followed by a breath‐hold (13.4 seconds) at maximal inspiration. Participants were coached in the inhalation scheme before entering the MRI scanner, and instructions were reiterated before each gas inhalation session to ensure compliance with breathing maneuvers. All gas inhalations were performed according to verbal breathing instructions provided by the attending MR radiographer. The PFP/oxygen gas mixture was administered from a 25‐L reservoir bag with plastic tubing combined with a manually operated three‐way gas switch, non‐rebreathe valve, and mouthpiece (Hans Rudolf, Shawnee, Kansas).

An FID whole‐lung spectroscopy scan (Table 2) was acquired at the onset of breath‐hold during the first gas inhalation session, allowing measurement of PFP’s ‐CF_3_^19^F resonant frequency. A 3D spoiled gradient‐echo sequence (Table 2) was acquired at the onset of breath‐hold for the remaining four gas inhalation sessions, enabling acquisition of four 3D ^19^F‐MR ventilation images per participant. Each ^19^F‐MRI acquisition was separated by an interval of approximately 6 minutes (mean [SD] = 358 [74] seconds), ensuring substantial gas washout from the lungs between acquisitions. Heart rate and oxygen saturations were monitored throughout using an MR‐compatible pulse oximeter (Nonin Medical, Plymouth, MA). A separate supply of oxygen was available in the event of clinically significant desaturation (in which a sustained oxygen saturation below 88% would be considered an adverse event), and a clinician was present on site throughout all MRI scans.

### 2.3 Image analysis

**FIGURE 2:**
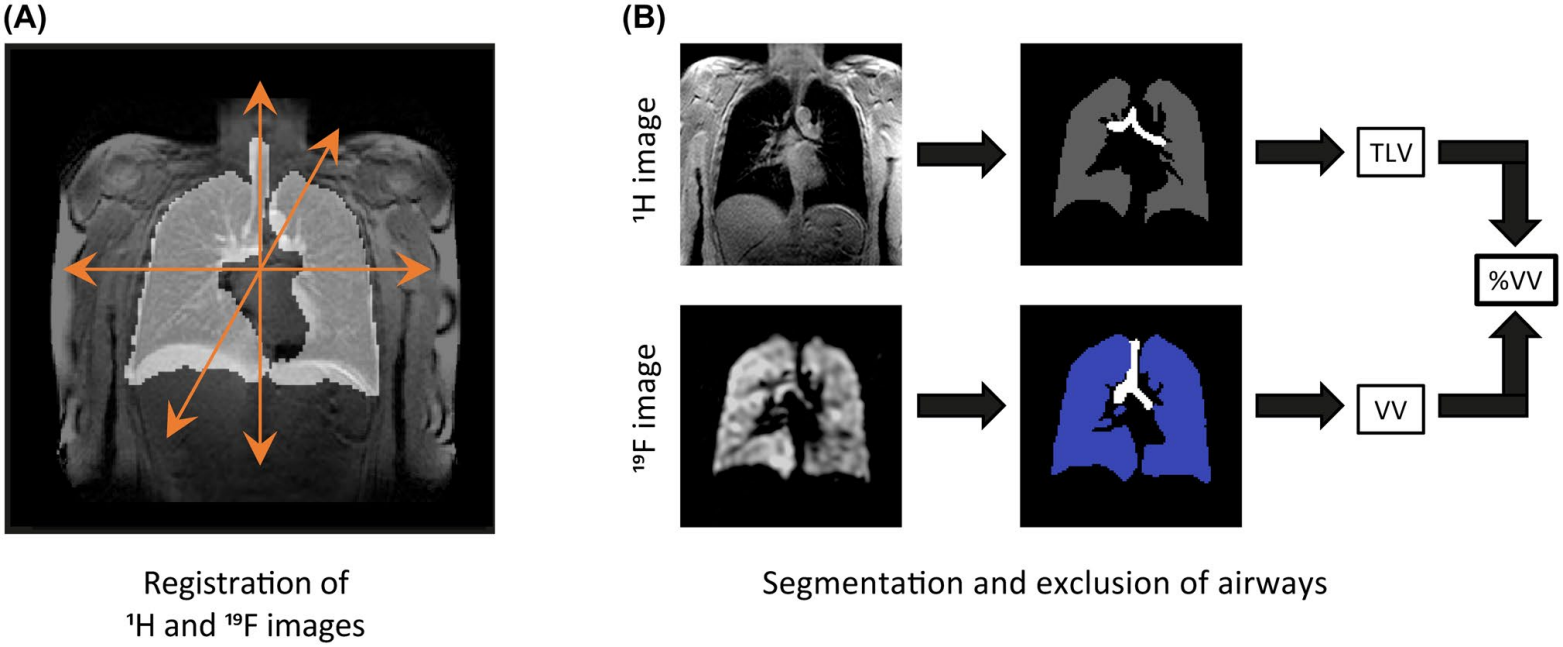
A, Registration of ^1^H images to ^19^F images, enabling correction of potential anatomical misalignment between scans. B, Semi‐automated segmentation of individual ^1^H and ^19^F image slices, performed independently by two observers. The percentage ventilated lung volume (%VV) was calculated for each of the four ventilation images per participant by dividing the ventilated lung volume (VV) by the total lung volume (TLV)

The MR images were analyzed independently by two observers (B.P., 3 years of MRI experience; M.N., 7 years of MRI experience) using in‐house software^35^ developed in *MATLAB* (MathWorks, Natick MA) combined with open‐source 3D segmentation functionality in *ITK‐SNAP*.^36^ The ^1^H‐MR and ^19^F‐MR image masks (depicting the anatomical boundaries of the lungs and PFP gas signal, respectively) were generated using *ITK‐SNAP*’s semi‐automated region‐growing algorithm, allowing rigid registration of ^1^H images to each of the four corresponding ^19^F‐MR ventilation images. Registered images were subsequently segmented (Figure 2) to calculate total lung volumes (from ^1^H images) and ventilated lung volumes for each ^19^F‐MR image, using a semi‐automated approach as previously described.^35^ Distinction between ventilated and nonventilated lung regions determined by the algorithm was assessed qualitatively by each observer and corrected manually where necessary, such as to amend segmentation regions where the automatic segmentation process was judged to have misassigned a region. This also included the removal of trachea, main bronchi, and major vessels from regions assigned as lung volume and ventilated lung volume. The value of %VV was subsequently calculated for each segmented image pair by dividing the ventilated volume from each ^19^F‐MR ventilation image by the total lung volume determined from the corresponding ^1^H image.

The SNR was measured for each ventilation image by placing a 4 × 4 cm^2^ region of interest in the apex of the right lung (signal) and a 100‐cm^2^ region of interest below the lung (noise) in a central image slice, where the trachea was seen to bifurcate. The SNR was calculated by applying the following expression in *MATLAB*, accounting for the Rayleigh distribution of background noise in magnitude images as follows^37^:

$$\left(\frac{\text{mean signal - mean noise}\;}{\text{standard deviation of noise}}\right)\sqrt{2‐\frac{\pi }{2}}.$$

### 2.4 Statistical analysis

The data were analyzed by fitting linear random‐effects models with three independent variance components: differences between participants, differences between acquisitions within each participant, and differences between observers within each acquisition. This permitted an estimation of the contribution of each individual component to the total variation from the true %VV. The models were fitted in R^38^ using the package lme4^39^ and the lmer command (with formula = %VV ~ 1 participant/acquisition). Estimates obtained from these fits were used to compute intraclass correlation coefficients (ICCs) with confidence intervals found using the bootstrap. Coefficients of variation were computed as the SD divided by the mean over all repeated %VV measurements, expressed as a percentage. Interobserver agreement in the calculated %VV values was further evaluated using the Dice similarity coefficient as a measure of spatial overlap between segmented image pairs. Comparison of SNR and %VV measurements between the two study sites were assessed using independently sampled t‐tests (95% confidence interval).

## 3 RESULTS

Participant demographic information is summarized in Table 3. The ^19^F‐MRI scans were well tolerated throughout by all participants, with no adverse events. Of the 40 participants initially recruited to the study, a total of 38 were included for image analysis; 2 participants attending study site B were excluded from analysis as a result of poor compliance with breathing instructions during one or more PFP gas inhalation sessions, which affected the ability to make a true assessment of ventilated lung volumes in these participants.

**TABLE 3:** Summary of participant demographics

Parameter		Site A (*n* = 20)	Site B (*n* = 18)	Combined (*n* = 38)
Sex	Male	11	9	20
	Female	9	9	18
Age (years)	Male	35 (23‐58)	43 (28‐64)	39 (23‐64)
	Female	43 (27‐67)	42 (26‐56)	43 (26‐67)
	Total	39 (23‐67)	43 (26‐64)	41 (23‐67)
Body mass index (kg/m^2^)		24.4 (18.3‐32.3)	23.3 (17‐34.9)	23.9 (17‐34.9)
Spirometry^a^	FEV_1_ (% pred.)	105 (88‐120)	100 (83‐114)	103 (83‐120)
	FVC (% pred.)	108 (86‐125)	100 (79‐117)	104 (79‐125)
	FEV_1_/FVC (%)	80 (71‐93)	81 (71‐96)	80 (71‐96)
Mean heart rate (bpm)^b^	Pre‐inhalation	70 (42‐104)	65 (48‐107)	68 (42‐107)
	Post‐inhalation	72 (44‐103)	67 (48‐108)	70 (44‐108)
Mean oxygen saturation (%)	Pre‐inhalation	98 (96‐100)	98 (96‐100)	98 (96‐100)
	Post‐inhalation	98 (95‐100)	98 (93‐99)	98 (93‐100)

Data are presented as mean values with range in parenthesis.

^a^ FEV_1_ = forced expiratory volume in 1 second; FVC = forced vital capacity; %pred. = percentage of predicted value.

^b^ bpm = beats per minute.

### 3.1 Value of %VV measurement reproducibility

Calculated %VV measurements for the remaining 38 study participants are presented in Table 4. The mean %VV value calculated for each participant was uniformly above 94% (range 94.0%‐99.5%, median 98.2%).

**TABLE 4:** Calculated %VV values for study participants (*N* = 38)

Participant	Observer 1					Observer 2				
	%VV 1	%VV 2	%VV 3	%VV 4	Mean %VV	%VV 1	%VV 2	%VV 3	%VV 4	Mean %VV
A1	98.0	96.9	98.6	97.4	97.7	98.9	97.2	98.9	97.8	98.2
A2	97.3	96.6	98.4	96.7	97.2	97.8	97.0	96.9	96.2	97.0
A3	98.9	98.8	98.9	98.9	98.9	98.9	98.7	97.7	98.6	98.5
A4	97.9	97.5	97.8	97.9	97.8	97.4	96.5	96.7	96.9	96.9
A5	98.6	98.2	98.8	98.8	98.6	99.7	99.2	99.1	99.1	99.2
A6	97.7	97.9	97.6	98.2	97.8	97.5	97.4	97.3	98.9	97.8
A7	98.8	99.1	98.5	98.3	98.7	98.6	98.3	98.9	97.9	98.4
A8	98.2	98.0	97.5	98.7	98.1	98.9	99.6	98.8	99.1	99.1
A9	98.1	97.8	97.7	98.1	97.9	97.9	97.4	97.1	98.1	97.6
A10	98.3	98.6	98.8	98.8	98.6	99.3	99.1	99.5	99.3	99.3
A11	97.8	97.8	98.1	98.5	98.1	97.8	97.4	98.0	97.7	97.7
A12	98.5	98.6	98.9	99.0	98.7	99.0	98.9	98.6	98.8	98.8
A13	98.5	98.7	99.1	98.9	98.8	98.9	99.2	99.0	98.9	99.0
A14	98.8	98.9	98.7	99.0	98.9	99.4	99.6	99.6	99.3	99.5
A15	97.6	97.8	98.3	98.3	98.0	97.7	97.9	97.9	97.6	97.8
A16	99.2	99.3	99.4	99.2	99.3	99.4	99.2	99.2	99.1	99.2
A17	98.9	98.9	99.1	99.1	99.0	98.3	98.5	97.3	98.4	98.1
A18	97.9	97.6	97.9	97.5	97.7	98.6	97.6	97.7	97.3	97.8
A19	98.7	97.3	97.8	99.2	98.3	97.8	96.4	97.6	97.7	97.4
A20	99.1	99.3	99.3	98.7	99.1	97.3	98.6	98.2	98.3	98.1
B1	96.4	96.6	96.3	95.4	96.2	94.3	94.1	96.1	97.1	95.4
B2	99.8	99.1	99.1	99.2	99.3	99.5	99.5	99.6	99.5	99.5
B3	98.1	98.5	98.2	98.5	98.3	97.6	98.3	97.6	97.2	97.7
B4	97.0	96.6	97.6	96.3	96.9	96.6	95.9	97.2	95.5	96.3
B5	98.4	98.1	98.4	98.7	98.4	98.2	97.6	98.2	97.8	97.9
B6	98.3	98.4	97.8	97.7	98.0	98.3	96.9	97.5	97.9	97.7
B7	99.4	99.2	99.6	99.8	99.5	98.5	98.5	98.3	98.9	98.5
B8	99.4	99.2	99.3	99.2	99.3	97.1	98.7	98.4	97.6	97.9
B9	99.3	98.4	99.0	97.6	98.6	96.9	97.0	96.1	96.4	96.6
B10	99.3	99.1	99.1	99.4	99.2	99.0	98.2	98.6	98.2	98.5
B11	98.0	98.0	97.7	98.0	97.9	97.6	97.2	97.2	97.3	97.3
B12	98.9	98.8	99.1	99.2	99.0	98.7	97.3	98.6	97.8	98.1
B13	93.8	97.3	97.0	98.4	96.6	90.4	93.2	95.3	96.9	94.0
B14	97.8	97.6	93.6	95.3	96.1	96.3	97.8	90.2	95.8	95.0
B15	97.6	98.7	99.1	98.9	98.5	98.1	98.7	98.0	99.0	98.4
B16	98.9	99.1	98.9	99.0	99.0	98.9	97.7	98.1	98.4	98.3
B17	99.3	99.4	99.4	99.2	99.3	98.2	97.7	98.3	98.6	98.2
B18	96.1	95.7	95.4	97.1	96.1	98.6	97.3	95.6	97.2	97.2

The coefficient of variation was calculated as CoV_observer1_ = 0.43% and CoV_observer2_ = 0.63%. Separate analyses by the two observers revealed ICCs (95% confidence interval) of 0.683 (0.578, 0.837) for observer 1 and 0.614 (0.493, 0.784) for observer 2. The combined analysis of both observers gave components of variance for differences between participants, differences between acquisitions within each participant, and differences between observer within each acquisition of 0.90, 0.18 and 0.54, respectively. Consequently, for a given participant, the SD of the error in a single image would be √(0.18 + 0.54) = 0.85, such that 95% of single image estimates would be within ± 1.7% of the true value.

### 3.2 Interobserver agreement

**FIGURE 3:**
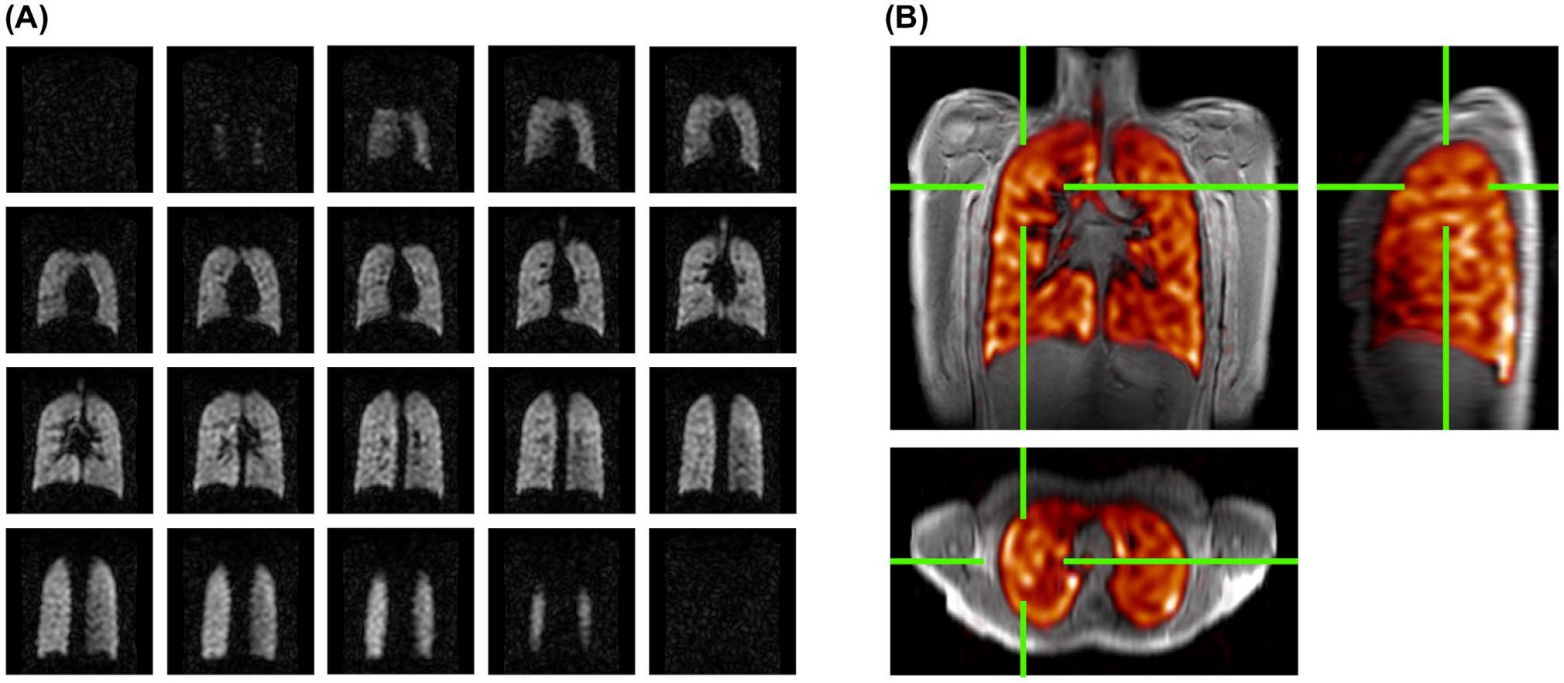
A, Representative ^19^F‐MR ventilation images (coronal views) from a healthy participant, acquired during a 13.4‐second breath‐hold scan following three deep breaths of a 79% PFP/21% oxygen gas mixture. The original magnitude image is displayed without any image processing or thresholding applied. An SNR of 13.5 was measured in a central lung slice of this image. B, Combined ^1^H and ^19^F‐MR ventilation images (colored, orthogonal views) in the same participant, showing homogeneous gas distribution throughout the lung fields

Figure 3A shows coronal slices from a representative 3D ^19^F‐MRI data set from 1 healthy participant (B2: 26‐year‐old female; forced expiratory volume in 1 second (FEV_1_) = 102% predicted, forced vital capacity [FVC] = 104% predicted, FEV_1_/FVC = 83%) acquired during a 13.4‐second breath‐hold at maximal inspiration. Figure 3B shows orthogonal views from the same participant, with ^19^F ventilation images (colormap) superimposed on the corresponding anatomical ^1^H MR images (grayscale). Homogeneous gas distribution can be visualized throughout the entire lung fields, with only minor apparent ventilation heterogeneity observed toward the most peripheral anterior–posterior lung slices.

The mean (SD) calculated %VV values across all analyzed participant data sets (*N* = 38) were similar between observers (98.2 [0.9]% and 97.8 [1.2]% for observer 1 and observer 2, respectively). The random effects model estimated the variation about the true %VV value due to observer variation as 0.54.

**FIGURE 4:**
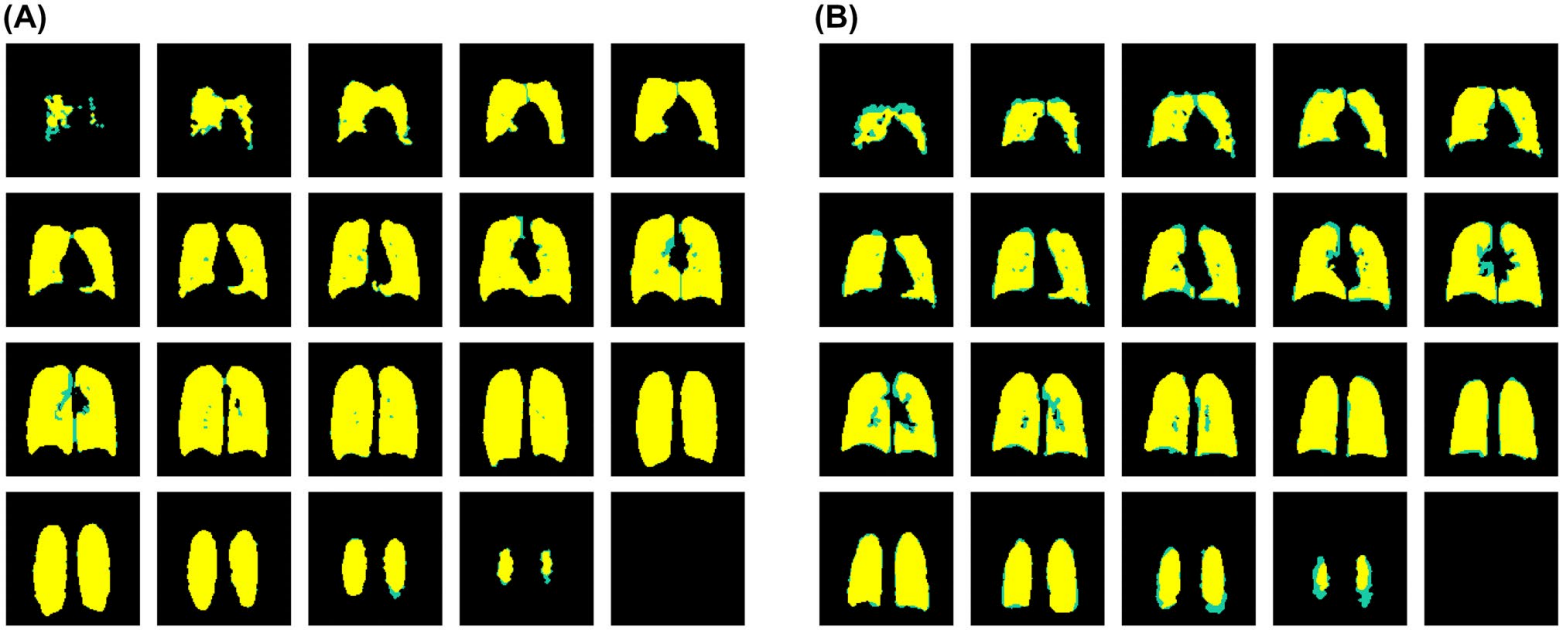
Combined ^19^F image segmentations performed independently by two observers, showing agreement (yellow) and disagreement (green) between respective segmentations. A, Example of close agreement in segmentations performed by the two observers (Dice similarity coefficient = 0.98), in which discrepancy is confined primarily toward the anterior slices. B, Example of greater discrepancy in segmentations performed by the two observers (Dice similarity coefficient = 0.93), in which more widespread differences are visible

Figure 4A shows a comparison of ^19^F‐MR image segmentations performed by the two observers for 1 participant (A7: 37‐year‐old male; FEV_1_ = 113% predicted, FVC = 120% predicted, FEV_1_/FVC = 76%). Interobserver agreement for respective image segmentations is shown in yellow, while disagreement between observers is shown in green. There is a high degree of spatial overlap between individual observer segmentations, with disagreement most prominent toward the peripheral lung slices. Figure 4B shows an equivalent combined segmentation data set from a different participant (A6: 34‐year‐old male; FEV_1_ = 113% predicted, FVC = 118% predicted, FEV_1_/FVC = 79%), in whom the discrepancy between observer segmentations (green) is more apparent. The Dice similarity coefficient, calculated as a measure of interobserver agreement across all ^19^F‐MR image segmentations performed, demonstrated a high mean (SD) value of 0.97 (0.02).

### 3.3 Comparison between sites

Image quality was of a sufficient standard across the two study sites to determine %VV values using the semi‐automated segmentation software, with comparable SNR achieved between the respective study sites (mean SNR_SiteA_ = 14.8 [2.5]; range = 8.4‐19.8; mean SNR_SiteB_ = 13.9 [4.0]; range = 6.5‐25.4). An independently sampled t‐test revealed no evidence of a difference in SNR values between study sites (*P* = .11).

The %VV measurements, averaged across both observers to provide a single value per participant, were compared between the two study sites. An independently sampled t‐test found no evidence of a difference in %VV values between the two study sites (*P* = .09), in which the mean %VV was 98.3 (0.6)% (range = 97.1‐99.3%) for site A, and 97.7 (1.3)% (range = 95.3‐99.4%) for site B, respectively.

### 3.4 Intraparticipant agreement

The SD around the mean of the four within‐participant %VV measurements were mean_observer1_ = 98.2% (SD = 0.4, range = 0.0‐1.7) and mean_observer2_ = 97.8% (SD = 0.5, range = 0.0‐2.9), measured by observers 1 and 2, respectively.

The spread of the four intraparticipant SNR measurements around the mean value were mean SNR_SiteA_ = 14.8 (SD = 1.2, range = 0.4‐2.3) and mean SNR_SiteB_ = 13.9 (SD = 1.2, range = 0.3‐2.5).

## 4 DISCUSSION AND CONCLUSIONS

This study investigated the ability of ^19^F‐MRI of inhaled PFP to provide quantitative measures of pulmonary ventilation, demonstrating good same‐day reproducibility of %VV measurements in a cohort of healthy participants. This work builds on a growing literature base surrounding the use of PFP as a potentially viable alternative to HP‐MRI for human ventilation imaging.

A goal of this study was to establish the technical feasibility of performing ^19^F‐MR ventilation imaging across different study sites, while maintaining reproducibility of data acquisition. We have successfully implemented a ^19^F‐MR imaging protocol to acquire ventilation images from 38 healthy participants at two research centers with sufficient image quality and SNR to determine %VV measurements. The %VV and its counterpart, ventilation defect percentage (equal to 100% minus %VV), are well‐established metrics within the HP‐MRI literature, providing quantitative volume‐independent measures that are sensitive to pathology and therapeutic response.^7^, ^8^ Our findings indicate a high degree of consistency in calculated %VV values across all study participants, with a low coefficient of variation between respective ^19^F‐MR acquisitions. It should be noted, however, that statistical assessment of %VV values in a cohort of healthy participants will inevitably be skewed toward the upper end of the %VV scale (and ventilation defect percentage calculations from the data would be equivalently biased).

The ICCs we report are slightly lower than values previously published for ^129^Xe and ^3^He.^7^, ^9^, ^11^ However, ICCs are determined by the values of the between‐acquisition and between‐observer components of variance relative to the between‐participant component of variance. There was little variation in %VV values measured in our cohort of healthy participants compared with previously reported studies,^7^, ^9^ with most of our participants demonstrating %VV values between 97% and 99%. As such, the relatively modest ICC values observed in our study likely reflect the marked homogeneity of our particular cohort, rather than diminished reproducibility per se. The similarity of within‐participant %VV values between observers (98.2% and 97.8% for observer 1 and 2, respectively), and the small intraparticipant SDs around these values (0.4 and 0.5, respectively), support the reproducibility of this technique. Moreover, the high degree of interobserver consistency and precision in calculated %VV values adds weight to the ability of our approach to provide reliable measures of pulmonary ventilation in a large group of healthy participants. Downstream application of these methods in patient cohorts will be beneficial in determining the capability of ^19^F‐MRI across a broader spectrum of ventilation defect severities.

Formal evaluation of the safety of inhaled PFP (eg, through a clinical trial of an investigational medicinal product) was beyond the scope of this study. Nonetheless, our work adds weight to the growing body of evidence surrounding the use of PFP for human ventilation imaging.^18^, ^19^, ^22^ To the best of our knowledge, this is the largest reported study to date involving ^19^F‐MRI of inhaled PFP. In total, 200 ^19^F‐MRI scans were performed on the 40 participants recruited to the study. Each acquisition consisted of three deep breaths of a 79% PFP/21% oxygen gas mixture. We observed no adverse events relating to gas inhalation, with no effect on participant heart rate or oxygen saturation (summarized in Table 3). This has implications for performing larger clinical studies, including future multicenter trials involving patients with respiratory disease, which will be crucial in establishing suitability of this approach for wider clinical use.

There were some limitations to our study. Notably, 2 participants were excluded from image analysis due to poor compliance with breathing instructions, which affected the quality of acquired ventilation images. In both of these cases, at least one of the four gas inhalation sessions was characterized by a failure to achieve maximal inspiration, which did not comply with the coached protocol. This resulted in images with a dearth of signal in combination with a substantial reduction in measured lung volume. Adherence to correct breathing instructions is crucial to maintaining reproducibility of image acquisitions. Importantly, our PFP inhalation protocol differs with HP‐MRI ventilation imaging, which typically involves a single inhalation of a small, fixed volume of gas. The sensitivity of inhaled PFP to changes in lung inflation level has previously been reported^40^ and underpins the specific breathing protocol used in this study (ie, three deep wash‐in breaths of gas, followed by a breath‐hold at maximum inspiration). This contrasts with the inhalation protocol reported by Couch et al, who used relaxed tidal wash‐in breaths, up to a cumulative 5 L, followed by a fixed (1 L) inhalation and breath hold.^18^ Nonetheless, by adopting this approach, the PFP wash‐in volume may be standardized relative to the volume of maximal inhalation achievable by each participant, rather than adopting a fixed wash‐in volume regardless of lung capacity. Our breathing protocol was therefore developed to achieve substantial replacement of air by PFP within the lungs, maximizing the SNR in ventilated regions and facilitating reproducibility of scan acquisitions. At the same time, the brevity of this wash‐in breathing protocol was designed to preserve the discernibility of poorly ventilated lung regions that is characteristic of patients with obstructive airways disease (data from pilot and ongoing studies not shown). Importantly, in the 38 participants who performed breathing maneuvers as instructed, we demonstrated good reproducibility of %VV measurements.

The use of advanced MR coil hardware (eg, array coils)^41^ may help to improve the overall SNR of ^19^F‐MR images, as well as address the spread of SNR values observed between participants. This may, in part, stem from differences in coil loading associated with varying body habitus, in addition to differences in breathing efficacy relating to the gas wash‐in protocol. Moreover, improved coil design may mitigate the tendency for spatially variable signal inhomogeneities (with slight signal dropoff particularly apparent toward the outermost anterior slices). Although this potentially reflects a degree of physiological ventilation heterogeneity, it is more likely the result of local field inhomogeneity arising from coil–scanner interaction. Segmentation of these particular slices does not, however, appear to have a substantial effect on global %VV measurements.

The ability to breathe PFP continuously over several respiratory cycles has recently been used as an alternative to static breath‐hold imaging,^22^ enabling dynamic image acquisition during free breathing. This approach plays to the strengths of ^19^F‐MRI of PFP compared with HP‐MRI, in which the thermally polarized gas does not exhibit the irrecoverable loss of signal over a dynamic imaging series that is unavoidable with hyperpolarized tracer gases (ie, ^3^He and ^129^Xe). Dynamic imaging reduces the requirement to follow a rigid inhalation protocol and offers potential advantages for imaging of patients with respiratory disease or younger children who may not be able to tolerate breath‐hold maneuvers. The repeatability of dynamic gas wash‐out measurements in patients with chronic obstructive pulmonary disease has recently been reported.^33^ Nonetheless, given the widespread use of %VV measurements in HP‐MRI literature, our study provides an important benchmark from which to determine the utility of static ^19^F‐MR ventilation imaging in preparation for performing future patient studies.

We report for the first time an evaluation of %VV measurements acquired by ^19^F‐MRI of inhaled PFP, demonstrating good same‐day reproducibility in a large number of healthy participants. The successful implementation of scan procedures across two different study sites provides a firm foundation from which to compare image quality and variability in patients with respiratory disease, including application to dynamic ventilation imaging.
